# Halogenated Diazabutadiene Dyes: Synthesis, Structures, Supramolecular Features, and Theoretical Studies

**DOI:** 10.3390/molecules25215013

**Published:** 2020-10-29

**Authors:** Valentine G. Nenajdenko, Namiq G. Shikhaliyev, Abel M. Maharramov, Khanim N. Bagirova, Gulnar T. Suleymanova, Alexander S. Novikov, Victor N. Khrustalev, Alexander G. Tskhovrebov

**Affiliations:** 1 M. V. Lomonosov Moscow State University, 1, Leninskie Gory, 119991 Moscow, Russia; 2 Department of Organic Chemistry, Baku State University, Z. Xalilov 23, Baku 1148, Azerbaijan; namiqst@gmail.com (N.G.S.); amaharramov@bsu.edu.az (A.M.M.); stella_stand@icloud.com (K.N.B.); gumusqiz91.sg@gmail.com (G.T.S.); 3 Saint Petersburg State University, Universitetskaya Nab. 7/9, 199034 Saint Petersburg, Russia; a.s.novikov@spbu.ru; 4 Peoples’ Friendship University of Russia, 6 Miklukho-Maklaya, 117198 Moscow, Russia; vnkhrustalev@gmail.com; 5 N.D. Zelinsky Institute of Organic Chemistry, Russian Academy of Sciences, 47 Leninsky Av., 119334 Moscow, Russia; 6 N.N. Semenov Federal Research Center for Chemical Physics, Russian Academy of Sciences, Kosygina 4, 119991 Moscow, Russia

**Keywords:** non-covalent interactions, crystal engineering, halogen bonding, azo dyes, DFT, QTAIM

## Abstract

Novel halogenated aromatic dichlorodiazadienes were prepared via copper-mediated oxidative coupling between the corresponding hydrazones and CCl_4_. These rare azo-dyes were characterized using ^1^H and ^13^C NMR techniques and X-ray diffraction analysis for five halogenated dichlorodiazadienes. Multiple non-covalent halogen···halogen interactions were detected in the solid state and studied by DFT calculations and topological analysis of the electron density distribution within the framework of Bader’s theory (QTAIM method). Theoretical studies demonstrated that non-covalent halogen···halogen interactions play crucial role in self-assembly of highly polarizable dichlorodiazadienes. Thus, halogen bonding can dictate a packing preference in the solid state for this class of dichloro-substituted heterodienes, which could be a convenient tool for a fine tuning of the properties of this novel class of dyes.

## 1. Introduction

Halogen bonding (XB) is one of the most intensively investigated areas in modern chemistry [1]. The field currently experiences a renaissance due to exploitation of such weak interactions for a number of functional applications, such as catalysis, drug design, nonlinear optics, reactivity control, and construction of functional supramolecular architectures [2,3,4,5,6,7,8,9,10] Utilization of non-covalent interactions lies at the foundation of the design supramolecular materials and control of their ultimate architectures [11,12,13,14]. XB has recently emerged as a powerful tool for the creation of such materials due to its stability, directionality and reversibility [15,16,17]. In this context, halogen-halogen interactions received particular attention and were intensively explored both experimentally and theoretically [18,19,20,21]. Arguably, XB can be more beneficial than the hydrogen bonding in the construction of functional materials and tuning their properties due to its higher directionality [10,22,23].

**Scheme 1:**
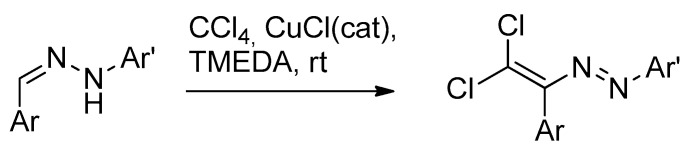
Copper-catalyzed synthesis of dichlorodiazadienes.

Recently, we discovered a novel class of azo-dyes, i.e., dichlorodiazadienes, which can be easily prepared via unprecedented copper-catalyzed reaction between CCl_4_ with *N*-substituted hydrazones (Scheme 1) [24]. Currently, very little is known about the chemistry and properties of these dichloro-substituted heterodienes [25,26,27,28,29,30,31].

Following our interest in construction of supramolecular architectures via non-covalent interactions [32,33,34,35,36,37,38,39] and chemistry of novel diazadienes, we report now the synthesis of halogenated dichlorodiazadienes to demonstrate that dichloro-substituted heterodiene fragment can behave as a strong XB donor/acceptor, what can be used in the design of heterodiene azo-dyes and their self-assembly in the solid state. Incorporation of a halogen atom(s) in the dichloro-dyes’ backbone completely changes the way the colorants self-assemble in the crystal. Thus, we show that the XB can dictate a packing preference in the solid state for this class of dichloro-substituted heterodienes. In addition, we performed DFT calculations and topological analysis of the electron density distribution within the formalism of Bader’s theory (QTAIM method), which support the presence of intermolecular non-covalent interactions halogen···halogen (Hal···Hal) in the solid state.

## 2. Results and Discussion

**Scheme 2:**
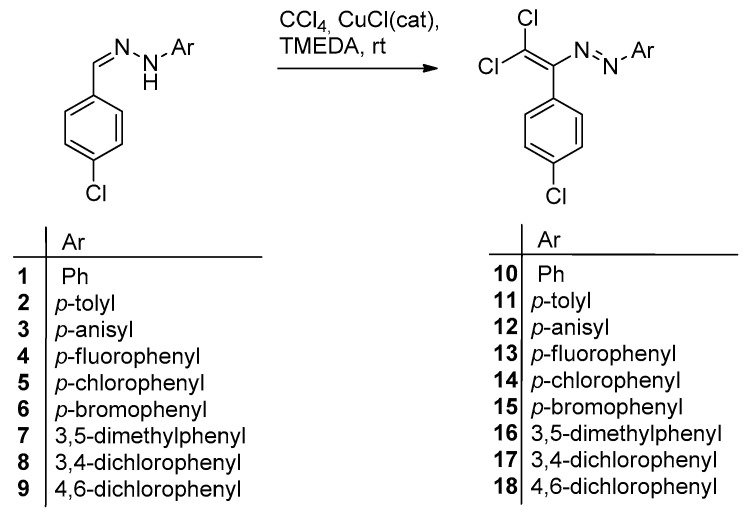
Copper-catalyzed synthesis of dichlorodiazadienes.

The target halogenated azabutadienes **10**–**18** were synthesized by Cu^I^-catalyzed reaction between the corresponding hydrazones **1**–**9** and CCl_4_ and isolated in up to 82% yield as red crystalline solids (Scheme 2).

**Figure 1:**
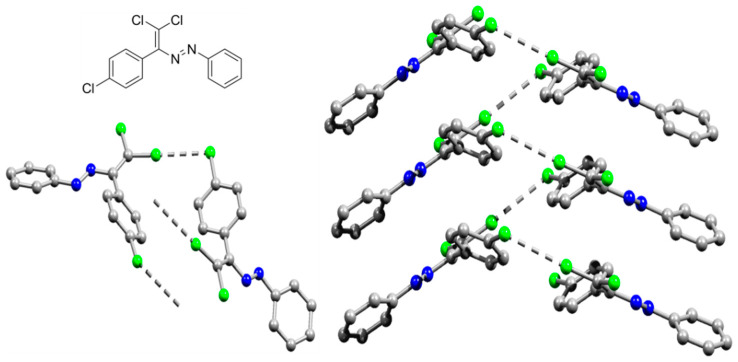
Ball-and-stick representation of **10** and its self-assembly via Cl···Cl bonding in the crystal. Blue, green and grey spheres represent nitrogen, chlorine, carbon atoms, respectively. Hydrogen atoms were omitted for clarity.

**Figure 2:**
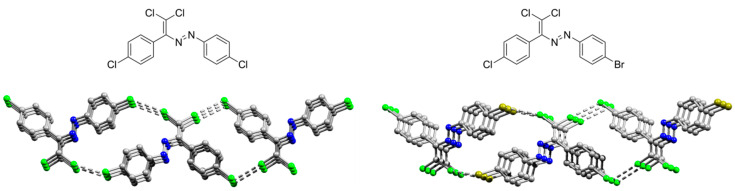
Ball-and-stick representations of **14** and **15** and their self-assembly via Cl···Hal bonding in the crystal. Olive-green, blue, green, and grey spheres represent bromine, nitrogen, chlorine, and carbon atoms, respectively. Hydrogen atoms were omitted for clarity.

**Figure 3:**
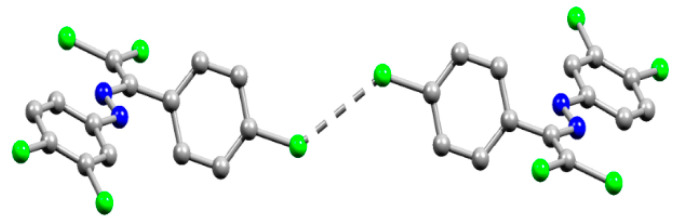
Ball-and-stick representation of **17** and its supramolecular dimerization via Cl···Cl type 1 bonding in the crystal. Blue, green, and grey spheres represent nitrogen, chlorine, and carbon atoms, respectively. Hydrogen atoms were omitted for clarity.

**Figure 4:**
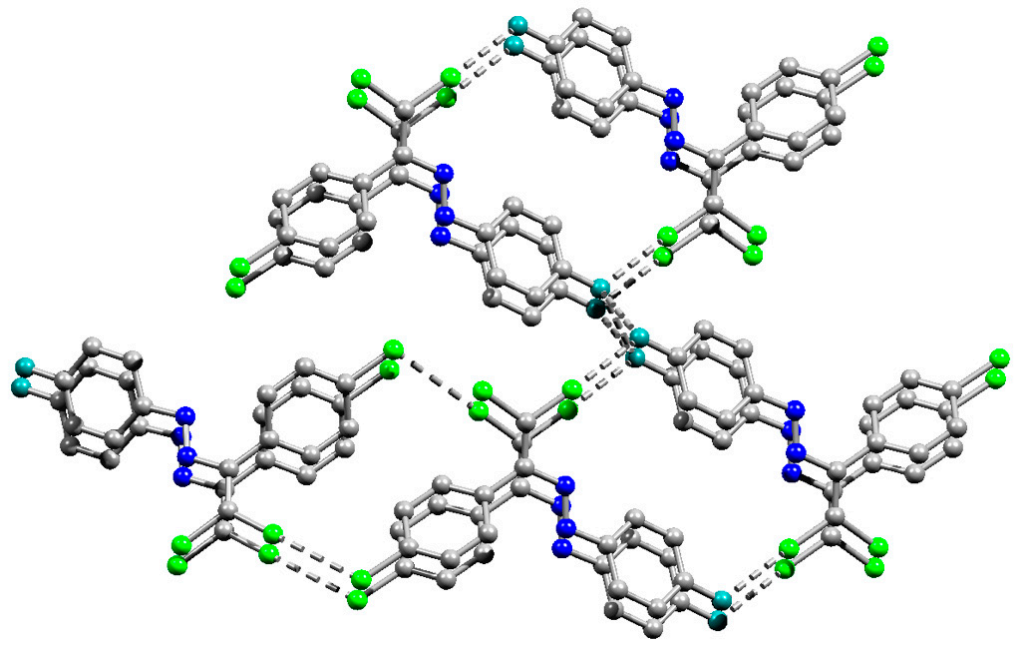
Ball-and-stick representation of **13** and its self-assembly via Cl···Cl bonding in the crystal. Blue, green, and grey and cyan spheres represent nitrogen, chlorine, carbon, and fluorine atoms, respectively. Hydrogen atoms were omitted for clarity.

The structure of **10**–**18** was confirmed by the ^1^H and ^13^C NMR spectroscopies and X-ray diffraction analysis for **10**, **13**–**15**, and **17** (Figure 1, Figure 2, Figure 3 and Figure 4). ^1^H NMR and ^13^C{^1^H} spectra (CDCl_3_) are consistent with their solid-state structures. Dyes **10**, **13**–**15**, and **17** could be easily recrystallized to produce large red crystals, suitable for analysis by single crystal X-ray crystallography. The structural investigations confirmed the formation of azabutadienes. Overall, metrical parameters for **10**, **13**–**15**, and **17** are similar to those reported for similar azabutadienes [26,29,30,31]. However, introduction of halogen atoms in the dichloro-dyes’ backbone has a dramatic impact on its self-assembly in the crystal. In the crystal packing of **10** (para-chloro substitution at the phenyl, attached the double C=C bond) dye molecules form shifted columns (Figure 1) via π-π interactions. The columns dimerize in the crystal via Cl···Cl attractive interactions between the neighboring dye molecules (type 2 contacts) [23]. The dichloroalkene acts as a donor of the halogen bond here (Figure 1).

Functionalization of dichloro-dyes with another extra halogen atom (compounds **14** and **15**) does not prevent the formation of columns and supramolecular dimerization via Cl···Cl interactions in the crystal (Figure 2). In addition to this, the columns in the crystal of **14** and **15** interact with another neighboring columns via Cl···Hal (Hal=Cl(**14**), Br(**15**)) type 2 bonding forming 3D supramolecular frameworks (Figure 2).

Introduction of one more halogen atom in the dichloro-dyes’ backbone completely changes its self-assembly in the crystal. Remarkably, crystal packing of **17** features only one type of Hal···Hal interaction between the chlorines of the p-cholorophenyl groups (Figure 3), which refer to repulsive type 1 contacts. Halogen atoms, attached to the alkene or dichlorobenzene moieties do not form any halogen bonding. Such a behavior is not very clear at the moment and requires additional studies. One plausible explanation is insufficient nucleophilicity of halogens in **17** for the formation of type 2 contacts.

Finally, when dichloro-dyes are functionalized with the fluorine atom (**13**, para- substitution at the phenyl, attached the double C=C bond, Figure 4), the situation with self-assembly in the crystal is similar to the brominated or chlorinated analogs **14** and **15**. The columns form 3D supramolecular frameworks via Cl···Cl and Cl···F type 2 contacts. An interesting peculiarity of self-assembly of **13** in the crystal is the formation of Cl···F type 1 contacts (Figure 4). Thus, the crystal structure of **13** features a bifurcated XB and a remarkable combination of type 1 and 2 halogen contacts (Figure 4).

**Figure 5:**
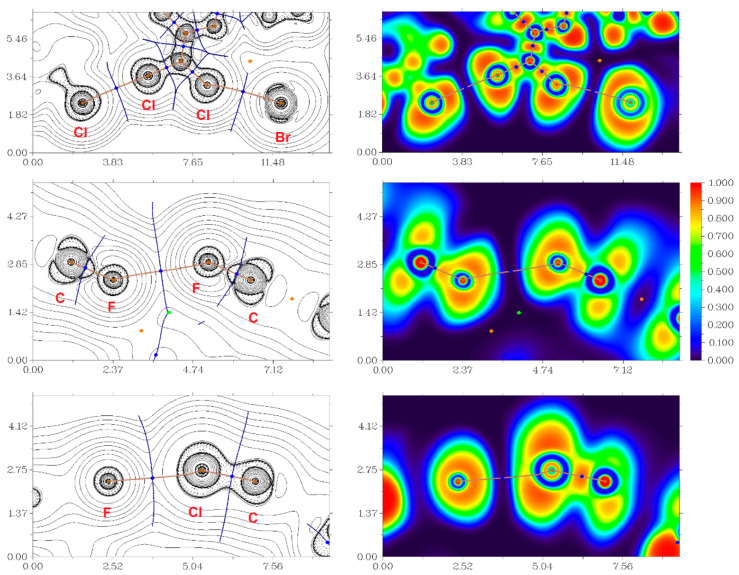
Contour line diagrams of the Laplacian of electron density distribution ∇^2^ρ(r), bond paths, and selected zero-flux surfaces (left) and visualization of electron localization function (ELF) analysis (right) for intermolecular Cl···Br and Cl···Cl contacts in **15** (top); F···F (center) and Cl···F (bottom) contacts in **13**. Bond critical points (3, −1) are shown in blue, nuclear critical points (3, −3)—in pale brown, ring critical points (3, +1)—in orange, cage critical points (3, +3)—in light green, length units—Å, bond paths are shown as pale brown lines, and the color scale for the ELF maps is presented in a.u.

Inspection of the crystallographic data suggests the presence of multiple intermolecular non-covalent interactions Hal···Hal in the crystals of **10**, **13**–**15**, and **17**. Indeed, the observed distances Hal···Hal are shorter than the sum of Bondi’s vdW radii for the corresponding atoms [40]. Thus, in addition to structural analysis, a detailed computational studies were desired. In order to understand the nature and quantify energies of various short halogen-halogen contacts the DFT calculations followed by the topological analysis of the electron density distribution within the QTAIM approach [41] were carried out at the ωB97XD/6-311++G ** level of theory for model supramolecular associates containing all types of these noncovalent interactions (see Computational details and Table S1 in the Supplementary Materials). Results of QTAIM analysis summarized in Table 1, the contour line diagrams of the Laplacian of electron density distribution ∇^2^ρ(r), bond paths, and selected zero-flux surfaces as well as visualization of electron localization function (ELF) analysis for selected short halogen-halogen contacts shown in Figure 5 for illustrative purposes.

The QTAIM analysis of **10**, **13**–**15**, and **17** demonstrates the presence of bond critical points (3, –1) for all weak contacts presented in Table 1. The low magnitude of the electron density (0.006–0.009 a.u.), positive values of the Laplacian of electron density (0.021–0.042 a.u.), and very close to zero positive energy density (0.001–0.002 a.u.) in these bond critical points (3, –1) are typical for halogen-halogen noncovalent interactions [5,39,43]. The balance between the potential and kinetic energy densities of electrons at the bond critical points (3, –1) for studied weak contacts in **10**, **13**–**15**, and **17** reveals that a covalent contribution is absent in these interactions [44] (Table 1). The Laplacian of electron density is typically decomposed into the sum of contributions along the three principal axes of maximal variation, giving the three eigenvalues of the Hessian matrix (λ_1_, λ_2_ and λ_3_), and the sign of λ_2_ can be utilized to distinguish bonding (attractive, λ_2_ < 0) weak interactions from non-bonding ones (repulsive, λ_2_ > 0) [45,46]. Thus, discussed noncovalent interactions in **10**, **13**–**15**, and **17** are attractive (Table 1). Overall, it follows from the results of theoretical calculations that all short halogen-halogen contacts in **10**, **13**–**15**, and **17** are very similar in terms of energies (their estimated strength per one contact vary from 1 to 3 kcal/mol), which correlates well with very close values of minimal and maximal electrostatic surface potentials on halogen atoms in isolated molecules **10**, **13**–**15**, and **17** (Figure S1 in the Supplementary Materials).

**Figure 6:**
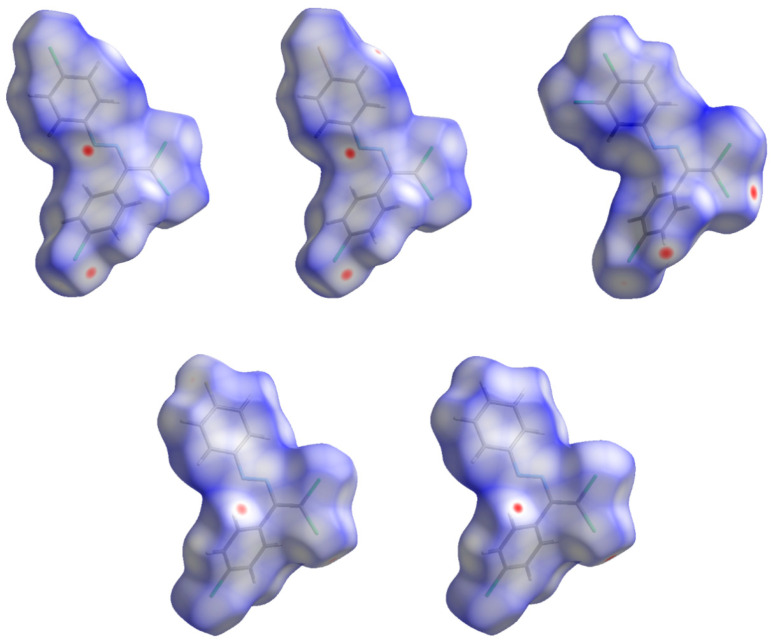
Visualization of Hirshfeld surfaces for X-ray structures **14**, **15**, and **17** (top), **13** and **10** (bottom).

To understand what kind of interatomic contacts give the largest contributions in crystal packing, we carried out the Hirshfeld surface analysis for all obtained X-ray structures **10**, **13**–**15**, and **17** (Table 2 and Figure 6). The Hirshfeld surface analysis for the X-ray structures **10**, **13**–**15**, and **17** reveals that in all cases crystal packing determined primarily by interatomic contacts involving chlorine and hydrogen atoms.

## 3. Materials and Methods

General remarks. Unless stated otherwise, all the reagents used in this study were obtained from the commercial sources (Aldrich, TCI-Europe, Strem, ABCR). NMR spectra were recorded on a Bruker Avance 300 (^1^H: 300 MHz, Karlsruhe, Germany); chemical shifts (δ) are given in ppm relative to TMS, coupling constants (J) in Hz. The solvent signals were used as references (CDCl_3_: *δ*_C_ = 77.16 ppm; residual CHCl_3_ in CDCl_3_: *δ*_H_ = 7.26 ppm; CD_2_Cl_2_: *δ*_C_ = 53.84 ppm; residual CHDCl_2_ in CD_2_Cl_2_: *δ*_H_ = 5.32 ppm); ^1^H and ^13^C assignments were established using NOESY, HSQC, and HMBC experiments; numbering schemes as shown in the Inserts. IR: Perkin-Elmer Spectrum One spectrometer (Waltham, MA, USA.), wavenumbers (*ṽ)* in cm^−1^. Mass-spectra were obtained on a Bruker micrOTOF spectrometer equipped with electrospray ionization (ESI) source (Bremen, Germany); MeOH, CH_2_Cl_2_, or MeOH/CH_2_Cl_2_ mixture was used as a solvent. Thermogravimetric analysis (TGA) and differential thermal analysis were determined using a Netzsch TG 209F1 Libra apparatus (Selb, Germany). Solvents were purified by distillation over the indicated drying agents and were transferred under Ar: Et_2_O (Mg/anthracene), CH_2_Cl_2_ (CaH_2_), hexane (Na/K). Flash chromatography: Merck Geduran^®^ Si 60 (Darmstadt, Germany) (40–63 μm).

The single point calculations based on the experimental X-ray geometries of **10**, **13**–**15**, and **17** have been carried out at the DFT level of theory using the dispersion-corrected hybrid functional ωB97XD [47] with the help of Gaussian-09 program package ([M. J. Frisch et al., Gaussian-09, Revision C.01, Gaussian, Inc., Wallingford CT, USA, 2010.], full citation for this program is given in the SI). The 6-311++G ** basis sets [48,49,50,51] were used for all atoms. The topological analysis of the electron density distribution with the help of the atoms in molecules (QTAIM) method developed by Bader [41] has been performed by using the Multiwfn program (version 3.6, Beijing, China) [52]. The Cartesian atomic coordinates for model supramolecular associates are presented in Table S1, Supporting Information. The Hirshfeld surfaces analysis has been performed by using the CrystalExplorer program (version 17.5, Perth, Australia) [53]. The normalized contact distances (d_norm_) [54] based on Bondi’s van der Waals radii [40] were mapped into the Hirshfeld surfaces.

### 3.1. Crystal Structure Determination

X-ray diffraction data for **10**, **13**–**15**, and **17** were collected at the ‘RSA’ beamline (λ = 0.80246 Å) of the Kurchatov Synchrotron Radiation Source. All datasets were collected at 100 K. In total, 720 frames were collected with an oscillation range of 1.0 in the *φ* scanning mode using two different orientations for each crystal. The semi-empirical correction for absorption was applied using the Scala program [55]**.** The data were indexed and integrated using the utility iMOSFLM from the CCP4 software suite [56,57]. For details, see Table S1. The structures were solved by intrinsic phasing modification of direct methods [58] and refined by a full-matrix least-squares technique on F^2^ with anisotropic displacement parameters for all non-hydrogen atoms. The hydrogen atoms were placed in calculated positions and refined within the riding model with fixed isotropic displacement parameters [*U*_iso_(H) = 1.5*U*_eq_(C) for the methyl groups and 1.2*U*_eq_(C) for the other groups]. All calculations were carried out using the SHELXTL program [59,60]**.**

Crystallographic data for **10**, **13**–**15**, and **17** have been deposited with the Cambridge Crystallographic Data Center, CCDC 2035010-2035014, respectively. Copies of this information may be obtained free of charge from the Director, CCDC, 12 Union Road, Cambridge CB2 1EZ, UK (fax: +44-1223-336033; e-mail: edeposit@ccdc.cam.ac.uk or www.ccdc.cam.ac.uk).

### 3.2. Synthetic Part

Schiff bases **1**–**9** were synthesized according to the reported method [20,21]. A mixture of (2-nitrophenyl)hydrazine (10.2 mmol), CH_3_COONa (0.82 g) and a corresponding 4-substituted aldehyde (10 mmol) were refluxed with stirring in ethanol (50 mL) for 2 h. The reaction mixture was cooled to room temperature and water (50 mL) was added to give a precipitate of crude product, which was filtered off, washed with diluted ethanol (1:1 with water) and dried in vacuo.

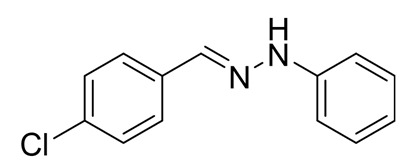
**1.** White solid (69%), mp 118 °C. ^1^H NMR (300 MHz, DMSO-*d*_6_) δ 10.46 (s, 1H, NH), 7.85 (s, 1H, CH), 7.66 (d, *J* = 8.4 Hz, 2H, arom), 7.43 (d, *J* = 8.4 Hz, 2H, arom), 7.23 (t, *J* = 7.7 Hz, 2H, arom), 7.09 (d, *J* = 7.9 Hz, 2H, arom), 6.76 (t, *J* = 7.2 Hz, 1H, arom). ^13^C NMR (75 MHz, DMSO-*d*_6_) δ 145.5, 135.4, 135.2, 132.5, 129.5, 129.1, 127.5, 119.41, 112.5.
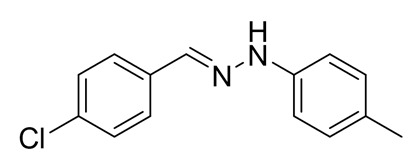
**2.** White solid (92%), mp 151 °C. ^1^H NMR (300 MHz, DMSO-*d*_6_) δ 10.33 (s, 1H, NH), 7.80 (s, 1H, CH), 7.66 (s, 1H, arom), 7.42 (d, *J* = 8.4 Hz, 2H, arom), 7.00 (q, *J* = 8.4 Hz, 5H, arom), 2.09 (s, 3H, CH_3_). ^13^C NMR (75 MHz, DMSO-*d*_6_) δ 138.6, 130.8, 130.1, 127.7, 125.4, 124.5, 123.3, 122.8, 107.92, 16.1.
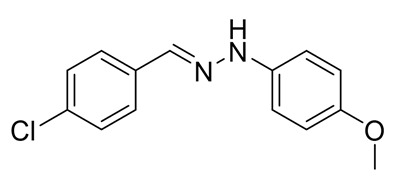
**3.** White solid (87%), mp 141 °C. ^1^H NMR (300 MHz, DMSO-*d*_6_) δ 10.24 (s, 1H, NH), 7.78 (s, 1H, CH), 7.63 (d, *J* = 8.5 Hz, 2H, arom), 7.41 (d, *J* = 8.5 Hz, 2H, arom), 7.01 (d, *J* = 8.9 Hz, 2H, arom), 6.84 (d, *J* = 8.9 Hz, 2H, arom), 3.69 (s, 3H, OCH_3_). ^13^C NMR (75 MHz, DMSO-*d*_6_) δ 153.2, 139.5, 135.5, 134.2, 132.1, 129.0, 127.3, 115.0, 113.5, 55.7.
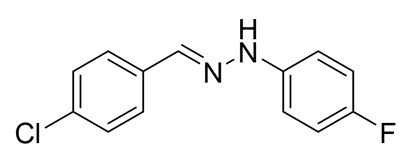
**4**. White solid (77%), mp 135 °C. ^1^H NMR (300 MHz, DMSO-*d*_6_) δ 7.02 (d, 2H, *J* = 6.0 Hz), 7.22 (t, 2H, *J* = 9.1 Hz), 7.37 (d, 2H, *J* = 9.1 Hz), 7.68–7.73(m, 2H), 7.87(s, 1H), 10.49 (s, 1H). ^13^C NMR (75 MHz, DMSO-*d*_6_) δ 114.3, 115.9, 116.2, 128.0, 132.1, 132,63, 136,7, 145.0, 109.9.
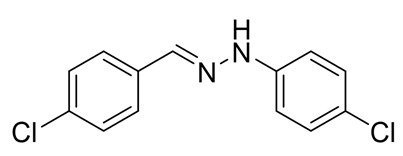
**5**. White solid (76%), mp 153 °C. ^1^H NMR (300 MHz, DMSO-*d*_6_) δ 10.58 (s, 1H, NH), 7.85 (s, 1H, CH), 7.67 (d, *J* = 8.3 Hz, 2H, arom), 7.48–7.38 (m, 2H, arom), 7.25 (d, *J* = 8.7 Hz, 2H, arom), 7.07 (d, *J* = 8.7 Hz, 2H, arom). ^13^C NMR (75 MHz, DMSO-*d*_6_) δ 144.4, 136.3, 135.0, 132.8, 129.3, 129.1, 127.7, 122.6, 113.9.
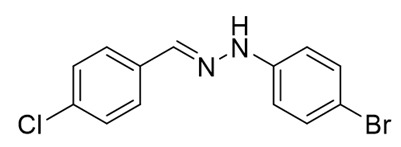
**6**. White solid (94%), mp 131 °C. ^1^H NMR (300 MHz, DMSO-*d*_6_) δ 10.59 (s, 1H, NH), 7.85 (s, 1H, CH), 7.67 (d, *J* = 8.5 Hz, 2H, arom), 7.43 (d, *J* = 8.5 Hz, 2H, arom), 7.37 (d, *J* = 8.8 Hz, 2H, arom), 7.03 (d, *J* = 8.8 Hz, 2H, arom). ^13^C NMR (75 MHz, DMSO-*d*_6_) δ 144.8, 136.4, 134.9, 132.8, 132.2, 129.1, 127.7, 114.4, 110.2, 39.9.
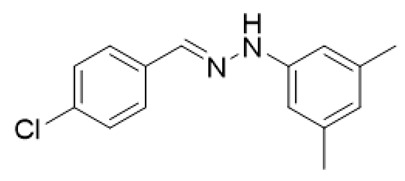
**7**. White solid (72%), mp 119 °C. ^1^H NMR (300 MHz, DMSO-*d*_6_) δ 10.30 (s, 1H, NH), 7.81 (s, 1H, CH), 7.65 (d, *J* = 8.5 Hz, 2H, arom), 7.42 (d, *J* = 8.5 Hz, 2H, arom), 6.69 (s, 2H, arom), 6.41 (s, 1H, arom), 2.22 (s, 6H, CH_3_). ^13^C NMR (75 MHz, DMSO-*d*_6_) δ 145.3, 138.5, 135.3, 135.0, 132.3, 129.1, 127.5, 121.3, 110.3, 21.7.
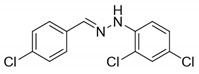
**8**. White solid (88%), mp 114 °C. ^1^H NMR (300 MHz, DMSO-*d*_6_) δ 10.16 (s, 1H, NH), 8.28 (s, 1H, CH), 7.69 (d, *J* = 8.7 Hz, 2H, arom), 7.56 (d, *J* = 8.7 Hz, 1H, arom), 7.51–7.43 (m, 3H, arom), 7.35–7.17 (m, 1H, arom). ^13^C NMR (75 MHz, DMSO-*d*_6_) δ 162.3, 140.9, 140.1, 134.6, 133.4, 129.2, 129.0, 128.5, 128.1, 122.8, 117.1, 115.5.
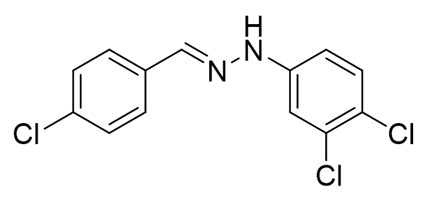
**9**. White solid (92%), mp 112 °C. ^1^H NMR (300 MHz, DMSO-*d*_6_) δ 10.74 (s, 1H, NH), 7.90 (d, *J* = 14.3 Hz, 1H), 7.69 (d, *J* = 8.3 Hz, 2H, arom), 7.44 (q, *J* = 8.3, 7.5 Hz, 3H, arom), 7.26 (s, 1H, CH), 7.00 (d, *J* = 8.4 Hz, 1H, arom). ^13^C NMR (75 MHz, DMSO-*d*_6_) δ 145.6, 137.6, 134.6, 133.2, 132.1, 131.5, 131.3, 130.2, 129.8, 129.1, 128.0, 120.1, 113.3, 112.8.

### 3.3. Synthesis of Dichlorodiazadiens

A twenty-milliliter screw neck vial was charged with DMSO (10 mL), **1**–**9** (1 mmol), tetramethylethylenediamine (TMEDA) (295 mg, 2.5 mmol), CuCl (2 mg, 0.02 mmol), and CCl_4_ (20 mmol, 10 equiv). After 3 h (until TLC analysis showed complete consumption of corresponding Schiff base) reaction mixture was poured into ~0.01 M solution of HCl (100 mL, ~pH = 2), and extracted with dichloromethane (3 × 20 mL). The combined organic phase was washed with water (3 × 50 mL), brine (30 mL), dried over anhydrous Na_2_SO_4_ and concentrated in vacuo. The residue was purified by column chromatography on silica gel using appropriate mixtures of hexane and dichloromethane (3/1–1/1).

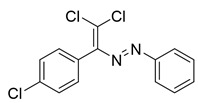
**10**. Red solid (73%), mp 85 °C. ^1^H NMR (300 MHz, CDCl_3_) δ 7.71–7.60 (m, 2H, arom), 7.35 (dd, *J* = 7.6, 3.8 Hz, 4H, arom), 7.28 (s, 1H, arom), 7.03 (d, *J* = 8.3 Hz, 2H, arom). ^13^C NMR (75 MHz, CDCl_3_) δ 134.6, 131.7, 131.3, 130.7, 129.4, 129.0, 128.4, 127.1, 126.2, 123.1.
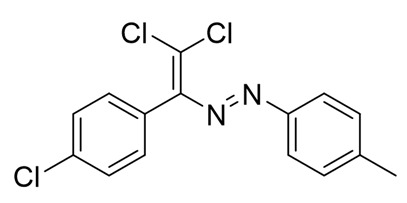
**11**. Red solid (79%), mp 90 °C. ^1^H NMR (300 MHz, CDCl_3_) δ 7.69 (d, *J* = 8.2 Hz, 2H, arom), 7.42 (d, *J* = 8.3 Hz, 2H, arom), 7.26 (d, *J* = 8.2 Hz, 2H, arom), 7.13 (d, *J* = 8.3 Hz, 2H, arom), 2.42 (s, 3H, CH_3_). ^13^C NMR (75 MHz, CDCl_3_) δ 162.3, 151.2, 150.9, 142.5, 134.7, 131.4, 131.0, 129.7, 128.4, 123.2, 21.5. Crystals, suitable for X-ray analysis, were obtained by the slow evaporation of saturated hexane/EtOAc (5/1) solution.
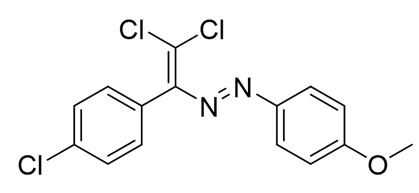
**12**. Red solid (72%), mp 96 °C. ^1^H NMR (300 MHz, CDCl_3_) δ 7.78 (d, *J* = 9.0 Hz, 2H, arom), 7.42 (d, *J* = 8.4 Hz, 2H, arom), 7.13 (d, *J* = 8.4 Hz, 2H, arom), 6.95 (d, *J* = 9.0 Hz, 2H, arom), 3.88 (s, 3H, OCH_3_). ^13^C NMR (75 MHz, CDCl_3_) δ 162.7, 162.3, 151.1, 147.2, 134.6, 131.4, 131.2, 128.4, 125.3, 114.2, 55.6.
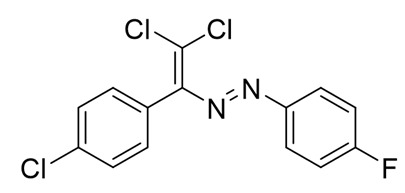
**13**. Red solid (68%), mp 77 °C. ^1^H NMR (300 MHz, CDCl_3_) δ 7.81 (dd, *J* = 8.6, 5.4 Hz, 2H), 7.43 (d, *J* = 8.3 Hz, 2H), 7.14 (t, *J* = 8.8 Hz, 4H). ^13^C NMR (75 MHz, CDCl_3_) δ 167.6, 166.4, 151.1, 149.3, 134.8, 131.4, 130.8, 129.6, 128.5, 125.4, 116.2, 115.9. Crystals, suitable for X-ray analysis, were obtained by the slow evaporation of saturated hexane/EtOAc (5/1) solution.
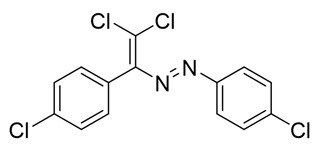
**14**. Red solid (67%), mp 94 °C. ^1^H NMR (300 MHz, CDCl_3_) δ 7.73 (d, *J* = 8.6 Hz, 1H), 7.43 (d, *J* = 8.4 Hz, 2H), 7.12 (d, *J* = 8.4 Hz, 1H). ^13^C NMR (75 MHz, CDCl_3_) δ 162.3, 151.1, 137.7, 136.5, 134.9, 131.4, 130.6, 129.3, 128.5, 124.4. Crystals, suitable for X-ray analysis, were obtained by the slow evaporation of saturated hexane/EtOAc (5/1) solution.
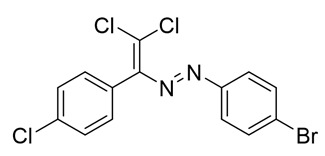
**15**. Red solid (70%), mp 105 °C. ^1^H NMR (300 MHz, CDCl_3_) δ 7.69–7.56 (m, 4H, arom), 7.49–7.39 (m, 2H, arom), 7.12 (d, *J* = 8.4 Hz, 2H, arom). ^13^C NMR (75 MHz, CDCl_3_) δ 151.4, 134.9, 132.3, 131.4, 130.6, 129.8, 128.5, 127.4, 126.3, 124.6. Crystals, suitable for X-ray analysis, were obtained by the slow evaporation of saturated hexane/EtOAc (5/1) solution.
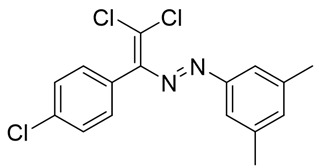
**16**. Red solid (82%), mp 145 °C. ^1^H NMR (300 MHz, CDCl_3_) δ 7.44 (d, *J* = 7.7 Hz, 4H, arom), 7.15 (d, *J* = 7.7 Hz, 3H, arom), 2.40 (s, 6H, CH_3_). ^13^C NMR (75 MHz, CDCl_3_) δ 157.7, 148.3, 146.7, 134.2, 130.2, 129.0, 126.8, 126.5, 123.9, 116.5, 16.6.
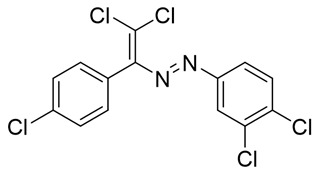
**17**. Red solid (66%), mp 115 °C. ^1^H NMR (300 MHz, CDCl_3_) δ 7.89 (s, 1H, arom), 7.68–7.61 (m, 1H, arom), 7.54 (d, *J* = 8.6 Hz, 1H, arom), 7.44 (d, *J* = 8.3 Hz, 2H, arom), 7.11 (d, *J* = 8.3 Hz, 2H, arom). ^13^C NMR (75 MHz, CDCl_3_) δ 151.5, 151.4, 135.7, 135.0, 133.5, 131.3, 130.8, 130.4, 129.8, 128.6, 124.5, 122.7. Crystals, suitable for X-ray analysis, were obtained by the slow evaporation of saturated hexane/EtOAc (5/1) solution.
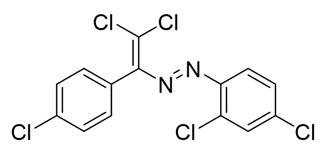
**18**. Red solid (71%), mp 121 °C. ^1^H NMR (300 MHz, CDCl_3_) δ 7.57 (d, *J* = 8.7 Hz, 1H, arom), 7.46 (d, *J* = 2.0 Hz, 1H, arom), 7.37 (d, *J* = 8.4 Hz, 2H, arom), 7.24 (d, *J* = 2.3 Hz, 1H, arom), 7.12 (d, *J* = 8.4 Hz, 2H, arom).

## 4. Conclusions

In summary, **9** novel halogenated dichlorodiazadienes were prepared and fully characterized, while for **5** of them single crystal structures were determined. Solid state structures contained multiple Hal⸱⸱⸱Hal interactions, which were studied by DFT calculations and topological analysis of the electron density distribution within the framework of Bader’s theory (QTAIM method). Calculations showed that the Hal⸱⸱⸱Hal interactions dictate a packing preference for this newly discovered class of dyes. These results further demonstrate the potential of Hal⸱⸱⸱Hal bonding in supramolecular engineering and crucial role in the stabilization of the intermolecular networks of dichlorodiazadienes. Further studies into photophysical properties of halogenated dichlorodiazadienes and their applications from our laboratory are underway and will be reported in due course.

## Figures and Tables

**Table 1:** Values of the density of all electrons—ρ(r), Laplacian of electron density—∇^2^ρ(r) and appropriate λ_2_ eigenvalues (with promolecular approximation), energy density—H_b_, potential energy density—V(r), and Lagrangian kinetic energy—G(r) (a.u.) at the bond critical points (3, −1), corresponding to various short halogen-halogen contacts in **10**, **13**–**15**, and **17**, and estimated energies for these interactions E_int_ (kcal/mol).

Halogen–Halogen Contact	ρ(r)	∇^2^ρ(r)	λ_2_	H_b_	V(r)	G(r)	E_int_^a^	E_int_^b^
**10** Cl···Cl, 3.377 Å (96% from the sum of Bondi’s vdW radii)	0.008	0.031	−0.012	0.002	−0.004	0.006	1.2	1.8
**13** F···F, 2.864 Å (97% from the sum of Bondi’s vdW radii)	0.006	0.031	−0.007	0.001	−0.006	0.007	≈2 *	≈2 *
**13** F···F, 2.917 Å (99% from the sum of Bondi’s vdW radii)	0.007	0.031	−0.008	0.001	−0.006	0.007	≈2 *	≈2 *
**13** Cl···F, 2.963 Å (92% from the sum of Bondi’s vdW radii)	0.009	0.042	−0.013	0.001	−0.008	0.009	2.5	2.7
**13** Cl···Cl, 3.405 Å (97% from the sum of Bondi’s vdW radii)	0.007	0.029	−0.011	0.002	−0.004	0.006	1.2	1.8
**14** Cl···Cl, 3.463 Å (99% from the sum of Bondi’s vdW radii)	0.007	0.026	−0.009	0.002	−0.003	0.005	0.9	1.5
**14** Cl···Cl, 3.399 Å (97% from the sum of Bondi’s vdW radii)	0.007	0.029	−0.011	0.002	−0.004	0.006	1.2	1.8
**15** Cl···Br, 3.637 Å (102% from the sum of Bondi’s vdW radii)	0.006	0.021	−0.008	0.001	−0.003	0.004	0.9	1.2
**15** Cl···Cl, 3.394 Å (97% from the sum of Bondi’s vdW radii)	0.007	0.030	−0.011	0.002	−0.004	0.006	1.2	1.8
**17** Cl···Cl, 3.469 Å (99% from the sum of Bondi’s vdW radii)	0.007	0.027	−0.009	0.002	−0.004	0.005	1.2	1.5

^a^ E_int_ = 0.49(−V(r)) (this correlation between the interaction energy and the potential energy density of electrons at the bond critical points (3, –1) was specifically developed for noncovalent interactions involving chlorine atoms) [42]. ^b^ E_int_ = 0.47G(r) (this correlation between the interaction energy and the kinetic energy density of electrons at the bond critical points (3, –1) was specifically developed for noncovalent interactions involving chlorine atoms) [42]. * There are no generally accepted specific correlations between the interaction energy and the potential or kinetic energy densities of electrons at the bond critical points (3, –1) for F···F noncovalent interactions, but it is clearly expected from values of V(r) and G(r) that strength of these contacts in **13** is approx. 2 kcal/mol.

**Table 2:** Main partial contributions of different interatomic contacts to the Hirshfeld surfaces of X-ray structures **10**, **13**–**15**, and **17**.

X-Ray Structure	Contributions of Different Interatomic Contacts to the Hirshfeld Surfaces
**10**	Cl-H 37.4%, H-H 21.0%, C-H 15.8%, C-C 7.9%, Cl-Cl 5.9%, N-H 5.8%, N-C 3.0%, -Cl-C 2.9%, Cl-N 0.1%
**13**	Cl-H 32.6%, H-H 16.2%, C-H 14.6%, C-C 7.4%, Cl-Cl 6.1%, N-H 5.8%, Cl-F 4.1%, N-C 3.1%, Cl-C 3.0%, F-H 2.8%, F-F 2.5%, F-C 1.7%
**14**	Cl-H 33.5%, H-H 17.1%, Cl-Cl 15.7%, C-H 12.6%, C-C 8.5%, N-H 5.2%, Cl-C 4.1%, N-C 3.2%, N-N 0.1%
**15**	Cl-H 30.7%, H-H 15.7%, C-H 13.0%, C-C 8.1%, Cl-Cl 5.8%, Br-Cl 5.7%, N-H 5.2%, Br-H 4.6%, Br-Br 3.8%, N-C 3.2%, Cl-C 2.2%, Br-C 1.8%, N-N 0.1%
**17**	Cl-H 44.6%, Cl-C 15.6%, H-H 10.5%, Cl-Cl 8.2%, C-H 8.1%, Cl-N 5.1%, C-C 4.4%, N-C 1.9%, N-H 1.5%

## Supplementary Materials

Figure S1. Visualization of electrostatic surface potentials for **10**, **13**–**15** and **17** with selected V_s,min_/V_s,max_ values (in kcal/mol). Table S1: Crystal data and structure refinement for **10**, **13**–**15** and **17**, Table S2. Values of the density of all electrons—ρ(r), Laplacian of electron density—∇^2^ρ(r) and appropriate λ_2_ eigenvalues (with promolecular approximation), energy density—H_b_, potential energy density—V(r), and Lagrangian kinetic energy—G(r) (a.u.) at the bond critical points (3, –1), corresponding to Cl···F halogen-halogen contacts in **13**, Table S3. Cartesian atomic coordinates for model supramolecular associates.
