# Supplementary material for: Halogenated Diazabutadiene Dyes: Synthesis, Structures, Supramolecular Features, and Theoretical Studies
Source: Molecules. 2020 Oct 29;25(21):5013. doi: 10.3390/molecules25215013 (PMC7663549; doi:10.3390/molecules25215013)
Supplement: Supplementary file 1 [file molecules-25-05013-s001.pdf]

Supporting Information

Halogenated Diazabutadiene Dyes: Synthesis, Structures, Supramolecular Features, and Theoretical Studies

Valentine G. Nenajdenko [1,*], Namiq G. Shikhaliyev [2], Abel M. Maharramov [2], Khanim N. Bagirova [2], Gulnar T. Suleymanova [2], Alexander S. Novikov [3], Victor N. Khrustalev [4,5] and Alexander G. Tskhovrebov [4,6,*]

[1] M. V. Lomonosov Moscow State University, 1, Leninskie Gory, Moscow 119991, Russia
[2] Department of Organic Chemistry, Baku State University, Z. Xalilov 23, Baku 1148, Azerbaijan; namiqst@gmail.com (N.G.S.); amaharramov@bsu.edu.az (A.M.M.); stella_stand@icloud.com (K.N.B.); gumusqiz91.sg@gmail.com (G.T.S.)
[3] Saint Petersburg State University, Universitetskaya Nab. 7/9, Saint Petersburg 199034, Russia; a.s.novikov@spbu.ru
[4] Peoples' Friendship University of Russia, 6 Miklukho-Maklaya, Moscow 117198, Russia; vnkhrustalev@gmail.com
[5] N.D. Zelinsky Institute of Organic Chemistry, Russian Academy of Sciences, 47 Leninsky Av., Moscow 119334, Russia
[6] N.N. Semenov Federal Research Center for Chemical Physics, Russian Academy of Sciences, Kosygina 4, Moscow 119991, Russia
* Correspondence: nenajdenko@org.chem.msu.ru (V.G.N.); alexander.tskhovrebov@chph.ras.ru (A.G.T.)

Table 1. Crystal data and structure refinement for **10**, **13-15** and **17**.

Identification code	**10**	**13**	**14**	**15**	**17**
Empirical formula	$C_{14}H_9N_2Cl_3$	$C_{14}H_8N_2FCl_3$	$C_{14}H_8N_2Cl_4$	$C_{14}H_8N_2Cl_3Br$	$C_{14}H_7N_2Cl_5$
Formula weight	311.58	329.57	346.02	390.47	380.47
Crystal size, mm	0.02 × 0.02 × 0.25	0.02 × 0.02 × 0.15	0.03 × 0.03 × 0.20	0.02 × 0.02 × 0.25	0.20 × 0.20 × 0.20
Crystal system	Monoclinic	Monoclinic	Monoclinic	Monoclinic	Monoclinic
Space group	$P2_1$/n	$P2_1$/n	$P2_1$	$P2_1$	$P2_1$/c
a, Å	9.960(2)	9.971(2)	9.988(2)	9.907(2)	14.183(3)
b, Å	3.9830(8)	4.0030(8)	3.9240(8)	3.9670(8)	8.4340(17)
c, Å	33.613(7)	33.996(7)	18.187(4)	18.371(4)	12.710(3)
α, deg.	90	90	90	90	90
β, deg.	93.63(3)	93.97(3)	97.35(3)	95.72(3)	91.35(3)
γ, deg.	90	90	90	90	90
V, Å^3	1330.8(5)	1353.7(5)	706.9(3)	718.4(3)	1519.9(6)
Z	4	4	2	2	4
Density (calc.), g/cm^3	1.555	1.617	1.626	1.805	1.663
Absorption coefficient, mm^{-1}	0.937	0.942	1.150	4.627	1.320
F(000)	632	664	348	384	760
θ range, deg.	2.37–31.40	2.36–30.97	2.32–30.97	2.33–30.94	1.62–31.00
Index ranges	$-12 \le h \le 12$<br>$-5 \le k \le 5$<br>$-43 \le l \le 43$	$-12 \le h \le 12$<br>$-5 \le k \le 5$<br>$-43 \le l \le 43$	$-12 \le h \le 12$<br>$-5 \le k \le 5$<br>$-23 \le l \le 21$	$-12 \le h \le 12$<br>$-5 \le k \le 5$<br>$-23 \le l \le 23$	$-18 \le h \le 18$<br>$-10 \le k \le 10$<br>$-16 \le l \le 15$
Reflections collected	15862	8581	8771	10999	13372
Independent reflections, R_{int}	2857, 0.115	2872, 0.091	3064, 0.087	3094, 0.050	3339, 0.049
Data / restraints / parameters	2171 / 0 / 173	2117 / 0 / 182	2876 / 1 / 182	3043 / 0 / 183	3144 / 0 / 191
Goodness-of-fit on F^2	1.048	1.018	1.066	1.015	1.070
Final R_1/wR_2 indices, I>2σ(I)	0.043 / 0.105	0.082 / 0.199	0.045 / 0.101	0.031 / 0.083	0.033 / 0.093
Final R_1/wR_2 indices (all data)	0.068 / 0.115	0.109 / 0.222	0.049 / 0.104	0.032 / 0.083	0.035 / 0.094
T_{min}, T_{max}	0.780, 0.980	0.860, 0.970	0.790, 0.950	0.360, 0.900	0.770, 0.770
Extinction coefficient	0.022(2)	0.021(2)	0.078(7)	0.087(8)	0.008(1)
$\Delta\rho_{max}$ / $\Delta\rho_{min}$,	0.428 / -	1.027 / -0.605	0.629 / -	0.597 / -0.406	0.330 / -

e·Å^{-3}	0.400		0.382		0.374

Additional DFT calculations within different levels of theory and dispersion corrections

We carried out additional DFT calculations in Orca 4.2.1 program package [WIREs Comput. Mol. Sci. 2012, 2, 73.] followed by the QTAIM analysis within different levels of theory and dispersion corrections (viz. PBE-D3BJ/6-311++G**, B3LYP-D3BJ/6-311++G** and M06-D3ZERO/6-311++G** levels of theory) for shortest and strongest Cl···F halogen-halogen contacts in **13** to check how the results of the QTAIM analysis can be affected by the kind of dispersion corrected functional employed. We found that values of the density of all electrons, Laplacian of electron density, energy density, potential energy density, and Lagrangian kinetic energy at the bond critical points (3, –1), corresponding to these short halogen-halogen contacts in **13** are almost independent on the dispersion corrected functional employed (**Table S2**).

Table S2. Values of the density of all electrons – ρ(**r**), Laplacian of electron density – $\nabla^2\rho(\mathbf{r})$ and appropriate λ_2 eigenvalues (with promolecular approximation), energy density – H_b, potential energy density – V(**r**), and Lagrangian kinetic energy – G(**r**) (a.u.) at the bond critical points (3, –1), corresponding to Cl···F halogen-halogen contacts in **13**.

Method/basis set (program)	ρ(**r**)	$\nabla^2\rho(\mathbf{r})$	λ_2	H_b	V(**r**)	G(**r**)
ωB97XD/6-311++G** (Gaussian 09)	0.009	0.042	-0.013	0.001	-0.008	0.009
PBE-D3BJ/6-311++G** (Orca 4.2.1)	0.009	0.041	-0.013	0.001	-0.008	0.009
B3LYP-D3BJ/6-311++G** (Orca 4.2.1)	0.009	0.042	-0.013	0.001	-0.008	0.009
M06-D3ZERO/6-311++G** (Orca 4.2.1)	0.009	0.042	-0.013	0.001	-0.008	0.009

Electrostatic surface potentials for 10, 13–15 and 17.

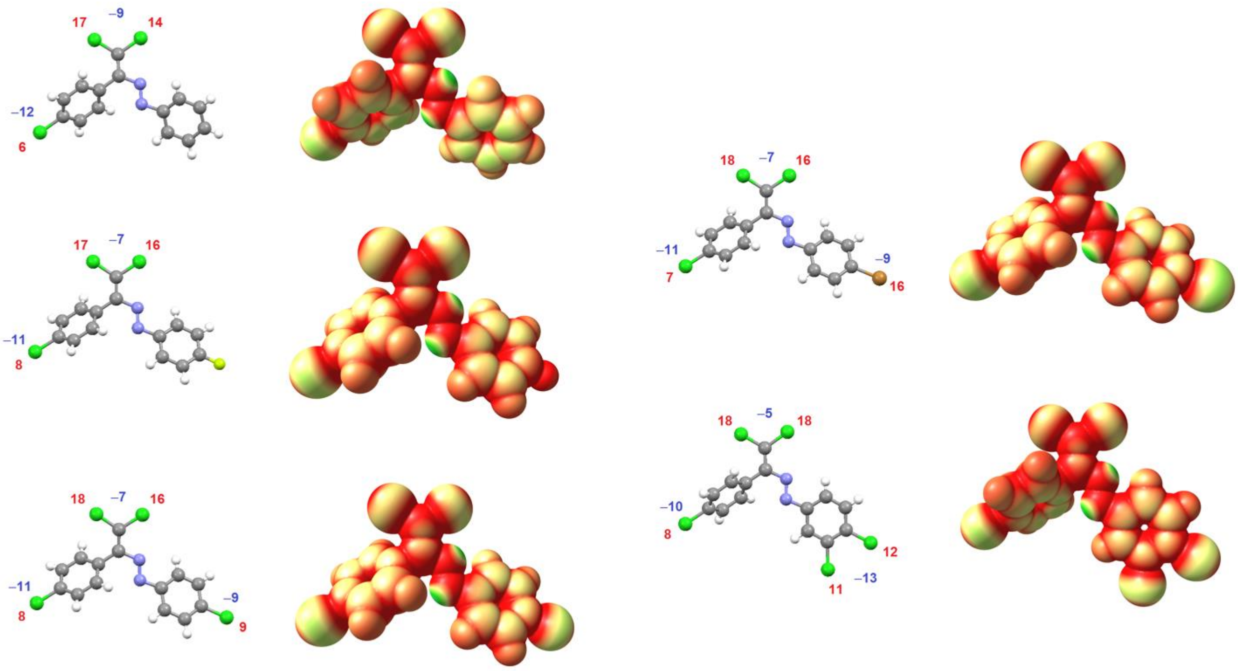

Figure S1. Visualization of electrostatic surface potentials for **10**, **13–15** and **17** with selected $V_{s,min}$/$V_{s,max}$ values (in kcal/mol).

Computational details

Gaussian-09 citation: M. J. Frisch, G. W. Trucks, H. B. Schlegel, G. E. Scuseria, M. A. Robb, J. R. Cheeseman, G. Scalmani, V. Barone, B. Mennucci, G. A. Petersson, H. Nakatsuji, M. Caricato, X. Li, H. P. Hratchian, A. F. Izmaylov, J. Bloino, G. Zheng, J. L. Sonnenberg, M. Hada, M. Ehara, K. Toyota, R. Fukuda, J. Hasegawa, M. Ishida, T. Nakajima, Y. Honda, O. Kitao, H. Nakai, T. Vreven, M. J. A.;, J. E. Peralta, F. Ogliaro, M. Bearpark, J. J. Heyd, E. Brothers, K. N. Kudin, V. N. Staroverov, T. Keith, R. Kobayashi, J. Normand, K. Raghavachari, A. Rendell, J. C. Burant, S. S. Iyengar, J. Tomasi, M. Cossi, N. Rega, J. M. Millam, M. Klene, J. E. Knox, J. B. Cross, V. Bakken, C. Adamo, J. Jaramillo, R. Gomperts, R. E. Stratmann, O. Yazyev, A. J. Austin, R. Cammi, C. Pomelli, J. W. Ochterski, R. L. Martin, K. Morokuma, V. G. Zakrzewski, G. A. Voth, P. Salvador, J. J. Dannenberg, S. Dapprich, A. D. Daniels, O. Farkas, J. B. Foresman, J. V. Ortiz, C. J.;, D. J. Fox, *Gaussian 09, Revision C.01*, Gaussian, Inc., Wallingford, CT, 2010.] program package.

Table S3. Cartesian atomic coordinates for model supramolecular associates.

Atom	X	Y	Z
**14**			
Cl	9.594875	-0.154998	5.402430
Cl	8.746953	0.750269	2.817287
Cl	5.671049	0.257807	10.771129
Cl	1.481100	4.080568	0.354799
N	6.274205	1.475816	4.005240
N	5.107497	1.748142	4.408741
C	7.134264	0.927241	4.980171
C	8.334513	0.558385	4.471511
C	6.763750	0.773813	6.412353
C	5.583182	0.125176	6.783927
H	5.003534	-0.215820	6.112929
C	5.245208	-0.026291	8.118706
H	4.443723	-0.473627	8.362213
C	6.087638	0.480298	9.090931
C	7.261090	1.151302	8.759039
H	7.825058	1.507993	9.435448
C	7.587544	1.286287	7.420652
H	8.389562	1.736762	7.180753
C	4.292836	2.323008	3.383846

C	4.809465	2.906507	2.220424
H	5.746674	2.930051	2.070712
C	3.940244	3.448804	1.293293
H	4.275105	3.854153	0.501444
C	2.565612	3.394652	1.527781
C	2.047081	2.819786	2.674970
H	1.109039	2.782116	2.815663
C	2.923431	2.299464	3.616531
H	2.585710	1.925899	4.422810
Cl	0.393125	-2.116998	-5.402430
Cl	1.241047	-1.211731	-2.817287
Cl	4.316951	-1.704193	-10.771129
Cl	8.506900	2.118568	-0.354799
N	3.713795	-0.486184	-4.005240
N	4.880503	-0.213858	-4.408741
C	2.853736	-1.034759	-4.980171
C	1.653487	-1.403615	-4.471511
C	3.224250	-1.188187	-6.412353
C	4.404818	-1.836824	-6.783927
H	4.984466	-2.177820	-6.112929
C	4.742792	-1.988291	-8.118706
H	5.544277	-2.435627	-8.362213
C	3.900362	-1.481702	-9.090931
C	2.726910	-0.810698	-8.759039
H	2.162942	-0.454007	-9.435448
C	2.400456	-0.675713	-7.420652
H	1.598438	-0.225238	-7.180753
C	5.695164	0.361008	-3.383846
C	5.178535	0.944507	-2.220424
H	4.241326	0.968051	-2.070712
C	6.047756	1.486804	-1.293293
H	5.712895	1.892153	-0.501444
C	7.422388	1.432652	-1.527781
C	7.940919	0.857786	-2.674970
H	8.878961	0.820116	-2.815663
C	7.064569	0.337464	-3.616531
H	7.402290	-0.036101	-4.422810
Cl	8.054459	-2.116998	12.635131
Cl	8.902382	-1.211731	15.220274
Cl	11.978285	-1.704193	7.266431
Cl	16.168234	2.118568	17.682762
N	11.375129	-0.486184	14.032320
N	12.541838	-0.213858	13.628820
C	10.515070	-1.034759	13.057390
C	9.314821	-1.403615	13.566049
C	10.885584	-1.188187	11.625208
C	12.066152	-1.836824	11.253634
H	12.645800	-2.177820	11.924631

C	12.404127	-1.988291	9.918855
H	13.205611	-2.435627	9.675348
C	11.561697	-1.481702	8.946630
C	10.388244	-0.810698	9.278521
H	9.824277	-0.454007	8.602113
C	10.061791	-0.675713	10.616908
H	9.259772	-0.225238	10.856808
C	13.356499	0.361008	14.653714
C	12.839869	0.944507	15.817137
H	11.902661	0.968051	15.966849
C	13.709091	1.486804	16.744268
H	13.374230	1.892153	17.536117
C	15.083722	1.432652	16.509779
C	15.602253	0.857786	15.362590
H	16.540296	0.820116	15.221897
C	14.725904	0.337464	14.421030
H	15.063625	-0.036101	13.614751
**15**			
Br	1.642172	4.159360	0.323548
Cl	9.766183	-0.192003	5.526267
Cl	8.935395	0.733102	2.946660
Cl	5.860453	0.209061	10.891857
N	6.465712	1.483658	4.140130
N	5.300892	1.753414	4.533323
C	7.324052	0.914790	5.105472
C	8.521194	0.538719	4.597301
C	6.952459	0.764838	6.540415
C	5.772755	0.103935	6.904178
H	5.198528	-0.235243	6.227835
C	5.433027	-0.061488	8.238583
H	4.635929	-0.517693	8.481701
C	6.276418	0.450651	9.214710
C	7.434078	1.128215	8.892990
H	7.992896	1.482071	9.574817
C	7.766979	1.284118	7.547617
H	8.561180	1.750240	7.313639
C	4.502515	2.350844	3.513325
C	5.030806	2.948274	2.363543
H	5.970659	2.972870	2.228274
C	4.178617	3.503654	1.425803
H	4.528876	3.926537	0.648923
C	2.807969	3.435025	1.628706
C	2.268888	2.837595	2.767521
H	1.327344	2.789198	2.889993
C	3.128453	2.318315	3.710744
H	2.777354	1.933516	4.505904
Br	8.264828	2.175860	-0.323548
Cl	0.140817	-2.175503	-5.526267

Cl	0.971605	-1.250398	-2.946660
Cl	4.046547	-1.774439	-10.891857
N	3.441288	-0.499842	-4.140130
N	4.606108	-0.230086	-4.533323
C	2.582948	-1.068710	-5.105472
C	1.385806	-1.444781	-4.597301
C	2.954541	-1.218662	-6.540415
C	4.134245	-1.879565	-6.904178
H	4.708472	-2.218743	-6.227835
C	4.473973	-2.044989	-8.238583
H	5.271071	-2.501194	-8.481701
C	3.630582	-1.532849	-9.214710
C	2.472922	-0.855285	-8.892990
H	1.914104	-0.501429	-9.574817
C	2.140021	-0.699382	-7.547617
H	1.345820	-0.233260	-7.313639
C	5.404485	0.367344	-3.513325
C	4.876194	0.964774	-2.363543
H	3.936341	0.989370	-2.228274
C	5.728383	1.520154	-1.425803
H	5.378124	1.943037	-0.648923
C	7.099031	1.451525	-1.628706
C	7.638112	0.854095	-2.767521
H	8.579656	0.805698	-2.889993
C	6.778547	0.334815	-3.710744
H	7.129646	-0.049984	-4.505904
Br	16.340844	2.175860	17.955980
Cl	8.216833	-2.175503	12.753261
Cl	9.047621	-1.250398	15.332868
Cl	12.122563	-1.774439	7.387671
N	11.517304	-0.499842	14.139398
N	12.682124	-0.230086	13.746205
C	10.658964	-1.068710	13.174056
C	9.461822	-1.444781	13.682227
C	11.030557	-1.218662	11.739113
C	12.210261	-1.879565	11.375350
H	12.784488	-2.218743	12.051693
C	12.549989	-2.044988	10.040945
H	13.347087	-2.501193	9.797827
C	11.706598	-1.532849	9.064818
C	10.548938	-0.855285	9.386538
H	9.990120	-0.501429	8.704711
C	10.216037	-0.699382	10.731911
H	9.421836	-0.233260	10.965889
C	13.480501	0.367344	14.766203
C	12.952210	0.964774	15.915985
H	12.012357	0.989370	16.051253
C	13.804399	1.520154	16.853725

H	13.454140	1.943037	17.630605
C	15.175047	1.451525	16.650822
C	15.714128	0.854095	15.512007
H	16.655672	0.805698	15.389535
C	14.854563	0.334815	14.568784
H	15.205662	-0.049984	13.773624
**17**			
Cl	1.063114	4.980193	7.554887
Cl	3.531773	4.732233	9.024772
Cl	0.308011	3.127074	1.313849
Cl	8.664569	-0.145065	3.645741
Cl	10.529217	0.646803	6.076743
N	4.527080	3.272898	6.704062
N	4.973549	2.654855	5.693516
C	3.212717	3.754648	6.518547
C	2.679921	4.412247	7.569245
C	2.479082	3.588498	5.234431
C	2.114526	2.318928	4.779285
H	2.325954	1.551856	5.298599
C	1.445125	2.172008	3.574076
H	1.186688	1.310644	3.268105
C	1.158452	3.300224	2.823251
C	1.519575	4.567854	3.249553
H	1.321628	5.329445	2.717914
C	2.171849	4.709124	4.459336
H	2.413671	5.574874	4.766198
C	6.308339	2.185418	5.878903
C	6.785087	1.367910	4.859336
H	6.225612	1.149554	4.123250
C	8.079991	0.871654	4.919692
C	8.897267	1.211544	5.994786
C	8.413681	2.030064	7.015624
H	8.973914	2.251878	7.749677
C	7.121750	2.518898	6.961495
H	6.790529	3.076723	7.655649
Cl	-1.063114	3.453807	-7.554887
Cl	-3.531773	3.701767	-9.024772
Cl	-0.308011	5.306926	-1.313849
Cl	-8.664569	8.579065	-3.645741
Cl	-10.529217	7.787197	-6.076743
N	-4.527080	5.161102	-6.704062
N	-4.973549	5.779145	-5.693516
C	-3.212717	4.679352	-6.518547
C	-2.679921	4.021753	-7.569245
C	-2.479082	4.845502	-5.234431
C	-2.114526	6.115072	-4.779285
H	-2.325954	6.882144	-5.298599
C	-1.445125	6.261992	-3.574076

H	-1.186688	7.123356	-3.268105
C	-1.158452	5.133776	-2.823251
C	-1.519575	3.866146	-3.249553
H	-1.321628	3.104555	-2.717914
C	-2.171849	3.724876	-4.459336
H	-2.413671	2.859126	-4.766198
C	-6.308339	6.248582	-5.878903
C	-6.785087	7.066090	-4.859336
H	-6.225612	7.284446	-4.123250
C	-8.079991	7.562346	-4.919692
C	-8.897267	7.222456	-5.994786
C	-8.413681	6.403936	-7.015624
H	-8.973914	6.182122	-7.749677
C	-7.121750	5.915102	-6.961495
H	-6.790529	5.357277	-7.655649
**13**			
Cl	-0.032395	0.721741	21.851064
Cl	0.748350	1.711283	19.283403
Cl	3.891256	1.144858	27.197673
F	7.769612	5.022964	17.151542
N	3.250101	2.449035	20.451076
N	4.415338	2.692018	20.858728
C	2.388694	1.855391	21.426794
C	1.194010	1.487515	20.926217
C	2.770253	1.686864	22.853913
C	3.954763	1.023167	23.213745
H	4.526264	0.680110	22.536135
C	4.297140	0.863847	24.544547
H	5.095342	0.405904	24.781270
C	3.478278	1.370227	25.525013
C	2.303027	2.063146	25.204183
H	1.751115	2.423416	25.886880
C	1.962424	2.213259	23.862189
H	1.168784	2.680809	23.631571
C	5.234088	3.310081	19.851130
C	4.706082	3.988990	18.752642
H	3.765936	4.058241	18.635976
C	5.569093	4.559017	17.840005
H	5.233544	5.028569	17.086087
C	6.916272	4.436525	18.039761
C	7.470453	3.773628	19.120274
H	8.412584	3.697171	19.222696
C	6.601585	3.226418	20.045460
H	6.943911	2.791692	20.816674
Cl	17.620711	11.287259	12.063361
Cl	16.839966	10.297718	14.631022
Cl	13.697060	10.864142	6.716752
F	9.818704	6.986036	16.762883

N	14.338215	9.559965	13.463348
N	13.172978	9.316982	13.055697
C	15.199622	10.153610	12.487630
C	16.394306	10.521485	12.988207
C	14.818063	10.322136	11.060511
C	13.633553	10.985833	10.700679
H	13.062052	11.328890	11.378289
C	13.291176	11.145153	9.369877
H	12.492974	11.603096	9.133155
C	14.110038	10.638773	8.389411
C	15.285289	9.945854	8.710242
H	15.837201	9.585584	8.027544
C	15.625892	9.795741	10.052235
H	16.419532	9.328191	10.282854
C	12.354228	8.698919	14.063294
C	12.882235	8.020011	15.161783
H	13.822380	7.950759	15.278448
C	12.019223	7.449983	16.074420
H	12.354772	6.980431	16.828337
C	10.672044	7.572475	15.874664
C	10.117863	8.235372	14.794150
H	9.175732	8.311829	14.691729
C	10.986731	8.782582	13.868965
H	10.644405	9.217308	13.097751
Cl	17.620711	7.284259	12.063361
Cl	16.839966	6.294718	14.631022
Cl	13.697060	6.861142	6.716752
F	9.818704	2.983036	16.762883
N	14.338215	5.556965	13.463348
N	13.172978	5.313983	13.055697
C	15.199622	6.150610	12.487630
C	16.394306	6.518485	12.988207
C	14.818063	6.319136	11.060511
C	13.633553	6.982833	10.700679
H	13.062052	7.325890	11.378289
C	13.291176	7.142153	9.369877
H	12.492974	7.600096	9.133155
C	14.110038	6.635773	8.389411
C	15.285289	5.942854	8.710242
H	15.837201	5.582584	8.027544
C	15.625892	5.792741	10.052235
H	16.419532	5.325191	10.282854
C	12.354228	4.695919	14.063294
C	12.882235	4.017011	15.161783
H	13.822380	3.947759	15.278448
C	12.019223	3.446983	16.074420
H	12.354772	2.977431	16.828337
C	10.672044	3.569475	15.874664

C	10.117863	4.232372	14.794150
H	9.175732	4.308829	14.691729
C	10.986731	4.779582	13.868965
H	10.644405	5.214308	13.097751
Cl	7.649711	7.284259	12.063361
Cl	6.868966	6.294718	14.631022
Cl	3.726060	6.861142	6.716752
F	-0.152296	2.983036	16.762883
N	4.367215	5.556965	13.463348
N	3.201978	5.313983	13.055697
C	5.228622	6.150610	12.487630
C	6.423306	6.518485	12.988207
C	4.847063	6.319136	11.060511
C	3.662553	6.982833	10.700679
H	3.091052	7.325890	11.378289
C	3.320176	7.142153	9.369877
H	2.521974	7.600096	9.133155
C	4.139038	6.635773	8.389411
C	5.314289	5.942854	8.710242
H	5.866201	5.582584	8.027544
C	5.654892	5.792741	10.052235
H	6.448532	5.325191	10.282854
C	2.383228	4.695919	14.063294
C	2.911235	4.017011	15.161783
H	3.851380	3.947759	15.278448
C	2.048223	3.446983	16.074420
H	2.383772	2.977431	16.828337
C	0.701044	3.569475	15.874664
C	0.146863	4.232372	14.794150
H	-0.795268	4.308829	14.691729
C	1.015731	4.779582	13.868965
H	0.673405	5.214308	13.097751
Cl	1.487369	-1.279759	29.020573
Cl	0.706624	-0.290217	31.588234
Cl	-2.436282	-0.856642	23.673964
F	-6.314638	3.021464	33.720095
N	-1.795127	0.447535	30.420560
N	-2.960364	0.690518	30.012909
C	-0.933720	-0.146109	29.444842
C	0.260964	-0.513985	29.945419
C	-1.315279	-0.314636	28.017724
C	-2.499788	-0.978333	27.657891
H	-3.071290	-1.321390	28.335502
C	-2.842166	-1.137653	26.327089
H	-3.640367	-1.595596	26.090367
C	-2.023304	-0.631273	25.346623
C	-0.848053	0.061646	25.667454
H	-0.296141	0.421916	24.984757

C	-0.507450	0.211759	27.009448
H	0.286190	0.679309	27.240066
C	-3.779114	1.308581	31.020507
C	-3.251107	1.987490	32.118995
H	-2.310962	2.056741	32.235660
C	-4.114119	2.557517	33.031632
H	-3.778570	3.027069	33.785550
C	-5.461297	2.435025	32.831876
C	-6.015479	1.772128	31.751362
H	-6.957610	1.695671	31.648941
C	-5.146611	1.224918	30.826177
H	-5.488937	0.790192	30.054963
**10**			
Cl	7.688933	0.751672	11.992874
Cl	6.819041	1.569660	14.595674
Cl	3.793041	1.229233	6.617869
N	4.374387	2.414096	13.391053
N	3.217827	2.682949	12.975088
C	5.243048	1.856078	12.416890
C	6.433090	1.451007	12.922086
C	4.882183	1.723046	10.977450
C	3.705285	1.063859	10.610461
H	3.130858	0.715347	11.281373
C	3.371052	0.914895	9.269310
H	2.576098	0.457647	9.020402
C	4.206383	1.438660	8.300849
C	5.368328	2.124134	8.636641
H	5.924172	2.494155	7.960362
C	5.699488	2.257963	9.979134
H	6.492875	2.719991	10.221333
C	2.388941	3.264467	13.980113
C	2.886343	3.934407	15.104225
H	3.823375	4.023627	15.233040
C	2.003216	4.464545	16.025386
H	2.333217	4.920598	16.789554
C	0.628914	4.331114	15.834177
H	0.026352	4.684406	16.477580
C	0.138293	3.688258	14.713084
H	-0.800033	3.603818	14.588965
C	1.015916	3.165290	13.768776
H	0.682148	2.743889	12.985487
Cl	6.186996	2.743172	4.779907
Cl	7.056888	3.561160	2.177107
Cl	10.082888	3.220733	10.154913
N	9.501542	4.405596	3.381728
N	10.658102	4.674449	3.797693
C	8.632881	3.847578	4.355891
C	7.442839	3.442507	3.850695

C	8.993746	3.714546	5.795331
C	10.170644	3.055359	6.162320
H	10.745071	2.706847	5.491409
C	10.504877	2.906395	7.503471
H	11.299830	2.449147	7.752380
C	9.669546	3.430160	8.471932
C	8.507600	4.115634	8.136141
H	7.951757	4.485655	8.812419
C	8.176441	4.249463	6.793647
H	7.383054	4.711491	6.551448
C	11.486988	5.255967	2.792668
C	10.989586	5.925907	1.668556
H	10.052554	6.015127	1.539741
C	11.872713	6.456045	0.747395
H	11.542712	6.912098	-0.016773
C	13.247015	6.322614	0.938605
H	13.849576	6.675906	0.295201
C	13.737636	5.679758	2.059698
H	14.675962	5.595318	2.183816
C	12.860013	5.156790	3.004005
H	13.193781	4.735389	3.787294
